# Contrast agent dose reduction in computed tomography with deep learning using a conditional generative adversarial network

**DOI:** 10.1007/s00330-021-07714-2

**Published:** 2021-02-25

**Authors:** Johannes Haubold, René Hosch, Lale Umutlu, Axel Wetter, Patrizia Haubold, Alexander Radbruch, Michael Forsting, Felix Nensa, Sven Koitka

**Affiliations:** 1 Department of Diagnostic and Interventional Radiology and Neuroradiology, University Hospital Essen, Hufelandstraße 55, 45147 Essen, Germany; 2 Institute for Artificial Intelligence in Medicine, University Hospital Essen, Essen, Germany; 3 Department of Diagnostic and Interventional Radiology, Kliniken Essen-Mitte, Essen, Germany; 4 Department of Neuroradiology, University Hospital Bonn, Bonn, Germany

**Keywords:** Image processing, computer-assisted, Tomography, spiral computed, Contrast media

## Abstract

**Objectives:**

To reduce the dose of intravenous iodine-based contrast media (ICM) in CT through virtual contrast-enhanced images using generative adversarial networks.

**Methods:**

Dual-energy CTs in the arterial phase of 85 patients were randomly split into an 80/20 train/test collective. Four different generative adversarial networks (GANs) based on image pairs, which comprised one image with virtually reduced ICM and the original full ICM CT slice, were trained, testing two input formats (2D and 2.5D) and two reduced ICM dose levels (−50% and −80%). The amount of intravenous ICM was reduced by creating virtual non-contrast series using dual-energy and adding the corresponding percentage of the iodine map. The evaluation was based on different scores (L1 loss, SSIM, PSNR, FID), which evaluate the image quality and similarity. Additionally, a visual Turing test (VTT) with three radiologists was used to assess the similarity and pathological consistency.

**Results:**

The −80% models reach an SSIM of > 98%, PSNR of > 48, L1 of between 7.5 and 8, and an FID of between 1.6 and 1.7. In comparison, the −50% models reach a SSIM of > 99%, PSNR of > 51, L1 of between 6.0 and 6.1, and an FID between 0.8 and 0.95. For the crucial question of pathological consistency, only the 50% ICM reduction networks achieved 100% consistency, which is required for clinical use.

**Conclusions:**

The required amount of ICM for CT can be reduced by 50% while maintaining image quality and diagnostic accuracy using GANs. Further phantom studies and animal experiments are required to confirm these initial results.

**Key Points:**

• *The amount of contrast media required for CT can be reduced by 50% using generative adversarial networks*.

• *Not only the image quality but especially the pathological consistency must be evaluated to assess safety*.

• *A too pronounced contrast media reduction could influence the pathological consistency in our collective at 80%*.

## Introduction

The use of iodine-based contrast media (ICM) is essential for dealing with various diagnostic tasks, such as the exclusion of pulmonary artery embolism or mesenteric ischemia. As the number of computed tomography (CT) scans continues to rise worldwide, the exposure of patients to ICM is increasing. The most important side effects of ICM include dose-dependent contrast-induced kidney injury (CIN) and thyroid dysfunction, as well as non-dose-dependent allergic reactions. As a result of the worldwide rise in life expectancy and the associated increase in overall morbidity and the rising prevalence of type II diabetes, the prevalence of chronic kidney disease (CKD) continues to grow [1]. Although it is now known that the risk of developing acute kidney injury (CI-AKI) in patients with reduced renal function after exposure to intravenous ICM has been overrated in the past, the actual risk of developing CI-AKI in patients with severe kidney disease remains unknown [2]. As radiologists, we still are increasingly faced with the challenge of making precise diagnostic statements for which we are frequently dependent on the intravenous administration of ICM and, on the other hand, we are confronted with a population with an increasing prevalence of CKD in whom the administration of intravenous ICM can potentially lead to a further deterioration in kidney function with all the consequences in terms of hospitalization, morbidity, and eventual mortality.

Through technical optimizations such as improved reconstruction procedures, and CT protocols with reduced tube voltage, significant savings in intravenous ICM have already been achieved in recent years [3]. Advances in deep learning–based image post-processing now open up possibilities for further ICM savings.

In the present study, we trained a generative adversarial network (GAN) to enhance ICM-induced image contrasts selectively and validated the results using dual-energy CT in which a 50% or 80% reduced ICM dose was simulated via the proportional subtraction of the calculated iodine maps.

## Materials and methods

### Ethics statement

This study was conducted in compliance with the guidelines of the Institutional Review Board of the University Hospital Essen (approval number 19-8904-BO). Due to the retrospective nature of the study, the requirement of written informed consent was waived by the Institutional Review Board. The data were completely anonymized before being included in the study.

### Computed tomography

A SOMATOM Force CT scanner (Siemens Healthineers AG) was used to perform dual-energy computed tomography with intravenous ICM. Reconstructions with a layer distance and a thickness of 1.5 mm were used. The scanning parameters were 0.25–0.5 s of rotation time, pitch 0.55–0.75, and 128 × 0.6 mm detector collimation. Dual-energy with a tube voltage of 80 kV and 150 kV was used. Furthermore, CareDose 4D was used to provide automatic tube current modulation adapted to the patient anatomy for effective mAs (Siemens Healthineers AG). The bolus tracking technique with an ROI placed in the aorta was used to acquire the arterial phase. Contrast media (1.5 ml/kg) were injected at a flow rate of 2.5–4.5 ml/s.

### Sequence reconstructions

The reconstructions of the virtual non-contrast (VNC) and isolated ICM sequence were performed in the dual-energy workflow of Syngo.via VB30 (Siemens Healthineers AG) employing a three-material mass fraction decomposition algorithm [4], which assumes that each voxel consists of fat, soft tissue, and iodine. As a result, the algorithm generates a map that encodes the iodine distribution in each CT voxel (isolated ICM sequence). In the next step, this distribution can be used to create VNC images. Both the isolated ICM sequence and VNC sequence were exported with a slice thickness and distance of 1.5 mm. Mixed images with a weighting factor of 0.5 with a slice thickness of 1.5 mm were reconstructed as the target images.

### Study design

**Fig. 1:**
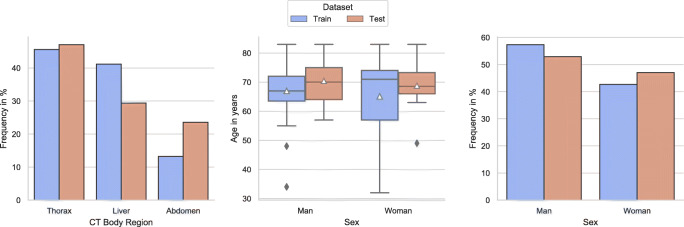
Distribution of the CT body region (left), age distribution within sex groups (middle), and frequency distribution for sex groups for the training (blue) and test (red) data (triangle indicates the mean)

The collected data contains 85 studies, including CT recordings from the abdomen, liver, and thorax during the arterial enhancement phase. Furthermore, the selected data were randomly split into an 80/20 collective for the training and testing process. The mean age of patients represented in the data was 66.8, with a standard deviation of 11.2 years. Patient age ranged from 32 to 83 years; 56% of the patients were men, and 44% were women. Figure 1 visualizes the distribution of the CT body regions, patient age, and patient sex for the training and testing subset. Table 1 illustrates the distribution of pathologies/findings in the test set.

**Table 1:** Distribution of pathologies/findings in the test set. Each examination containing the pathology/finding at least once was counted as one

Finding/pathology	Number of examinations containing the finding/pathology
Lung	
- Metastasis	2
- Nodule (<8 mm)	7
Liver	
- Cyst	7
- Hemangioma	2
- Metastasis	5
- HCC	2
Lymph node metastasis	3
Gastric cancer	2
Bone	
- Hemangioma	3
- Metastasis	2
- Bone island	5
Uterine myoma	1
Renal cyst	7
Adrenal adenoma	4

**Fig. 2:**
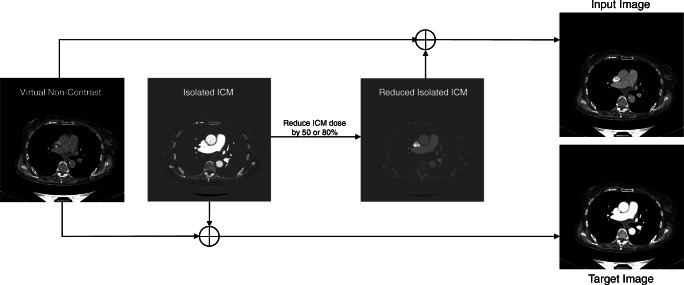
Schematic illustration of the generation of the used image pairs (input, target) through the combination of the VNC and the isolated ICM images

In general, each study contained two CT image series of interest: virtual non-contrast and isolated ICM. These allow us to combine both image types and generate the needed input images by adjusting the ICM dose to the desired level, as shown in Fig. 2.

**Fig. 3:**
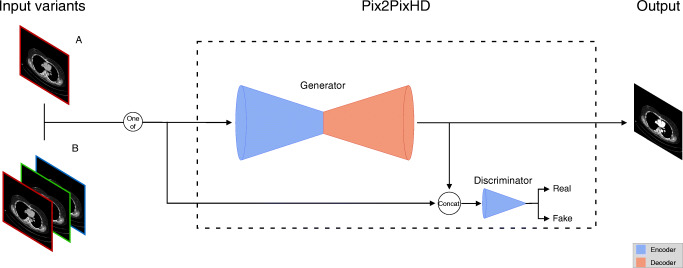
The schematic network architecture (blue = encoder, red = decoder) and the flow of the input data (2D (A) and 2.5D (B)) through the network, resulting in the final prediction with the enhanced variant of the input image

The CT image pairs used for training comprised the input image, which consisted of a dual-energy CT image reduced by 50% or 80% ICM, and the corresponding target image, which contained the original 100% ICM dose. A VNC image was combined with the reduced isolated ICM image to generate the input images (Fig. 2). In addition, the target image was created by combining the full dose isolated ICM image with the VNC image (Fig. 2). In addition to this standard procedure for generating the input images, there were two different experimental setups that were slightly different regarding the image input format. The first input variant 2D received one reduced ICM CT slice as the input image and predicted the corresponding 100% ICM image. The 2.5D model received three reduced ICM CT slices as input. Those three slices were mapped to the different channels of an RGB color image. More precisely, the slice corresponding to the target was placed on the middle channel, the previous slice on the first channel, and the subsequent slice on the last channel. The hypothesis was that by including neighborhood information, predictions could be stabilized at the cost of being unable to translate the very first and very last image slices. The flow of the input data through the network architecture is visualized in Fig. 3.

The model architecture used in this paper is very similar to the original Pix2PixHD model [5], a generative adversarial network for paired image-to-image translation tasks. In the reference implementation of Pix2PixHD, transposed convolutions are used as an upsampling method [5]. These, however, tend to generate checkerboard artifacts. Therefore, in order to reduce the risk of those artifacts, the standard upsampling method was replaced by the bilinear upsampling followed by a 3 × 3 convolution, which tends to generate fewer artifacts, which was shown by Odena et al and Wojna et al [6, 7].

In general, all images were loaded with an unchanged size of 512 × 512. Depending on the selected input type, the first convolution layer had between 1 and 3 input channels. In contrast, only one output channel was defined for each model. During training, only moderate data augmentation, in the form of horizontal flipping, was used. Additionally, the number of filters for the first convolutional layer was set to 32 and 64 for the generator and discriminator. Since neighbor slices contain very similar information, only every fifth slice of a volume was used for training in order to reduce training times. Each model was trained for up to 200 epochs, with weights being saved every 10 epochs for further analysis. An Adam optimizer was used for optimizing the model [5], with a batch size of 1, the learning rate set to constant 0.002 during the first 100 epochs and then linearly decaying to zero for the last 100 epochs, and a momentum (beta1) of 0.5. In addition, the feature matching loss weight was set to 10. All other network hyperparameters were set to the defaults of Pix2PixHD. Furthermore, for each model configuration, five models were trained with identical data to reduce the randomness in the performance of a single model. By simply averaging the predictions of these five models, an ensemble of GANs was created that produces empirically better-perceived image quality.

In the reference implementation of Pix2PixHD, input data is expected to be 8 bits per channel by default [5]. This prerequisite was an obstacle for the application within medical imaging and especially within CT images. Since CT images were captured in Hounsfield units (HU), the resulting theoretical value range is from −1024 to + 3071 HU for a 12-bit scanner quantization. Thus, the data loader in Pix2PixHD was extended to support loading 16-bit PNG images. Before feeding the images into the networks, the image data were normalized to a range from −1 to 1. It should be noted that for the volumes used, the HU range was limited to the theoretically possible maximum value range.

### Evaluation methods

Four different metrics and scores were used to evaluate the images generated by the different models. These were the L1 loss or mean absolute error (MAE), the structural similarity index (SSIM), the peak signal-to-noise ratio (PSNR), and the Fréchet inception distance (FID) [8]. The L1 loss indicates the mean absolute pixel difference between the generated and the target image. A low L1 value indicates a close approximation of the target images. In contrast, both the SSIM and the PSNR try to ensure a less superficial comparison between the generated and the target image [9]. The PSNR is based on the mean squared error (MSE)+ and approaches infinity [10]. This means that a rising PSNR value correlates with higher image quality.

In contrast, the SSIM provides a score based on factors derived from the human visual system (HVS) [10]. Instead of the classical error summation, each image difference is expressed by the three factors correlation loss, luminance distortion, and contrast distortion. An SSIM of 0 represents that the images have no correlation, whereas an SSIM of 1 means that the target image is identical to the prediction [9, 10].

Lastly, the FID uses activation vectors extracted from inception networks, which were pre-trained on ImageNet [8]. Therefore, both the real and the generated images are propagated through the network, but instead of using the final classification layer, the feature maps from the last pooling layer are used [8]. Subsequently, the mean and the covariance are calculated for all extracted feature maps of the two image domains (real and generated). These statistics can then be used to calculate the Fréchet distance. The lower the FID value, the better the image quality and similarity [8]. Within this study, the real CT volume is defined as the target domain, and the predicted volumes are defined as the source domain. For each volume pair, we calculated the FID value and used this value for further analysis. All the named scores and metrics have specific advantages and disadvantages for evaluating image similarities, but, combined, they provide an overall impression of the achieved image quality.

In addition to evaluating the metrics and scores, it is recommended to examine the generated images from a radiological perspective. For this reason, the generated images were also presented to three professional radiologists with 9, 4, and 3 years of experience using a visual Turing test (VTT) [11, 12]. This test was divided into two subtests: volume and slice. Within the volume test, artificially generated and non-artificially generated CT volumes were presented to the radiologists’ side by side. The radiologists’ task was to distinguish between the two volumes and to select the non-artificially generated one. This distinction is measured with the real/fake accuracy, which represents the percentage of cases where the radiologist selected the non-artificial CT as real. The lower the real/fake accuracy, the more difficult it is for a radiologist to distinguish between real and fake. During the slice test, the radiologists were asked to select the non-artificial slice without the ability to scroll through the complete image stack. The motivation for this was that Pix2PixHD is by design a two-dimensional model architecture that contains only very limited information about the entire body region captured during image acquisition.

For these tests, the volumes and slices were displayed within a DICOM viewer supporting standardized tools such as windowing, zooming, panning, and scrolling. This test was intended to determine whether the generated images not only provide good perceptual results but also withstand radiological criteria such as that the images should be diagnostically equivalent. Therefore, the radiologists were also asked whether the two slices or volumes were pathologically consistent. This leads to a second evaluation metric, the so-called pathological consistency, which indicates the number of cases where the non-artificial and artificial CT slices were pathological equivalent. Pathological consistency is intended to ensure that no pathology is visible in one examination that is not visible in the other. This evaluation metric was used to exclude that pathologies were accidentally inserted or removed. As opposed to the real/fake accuracy, a low pathological consistency indicates that the artificially created slices or volumes are not diagnostically equivalent in comparison to the real slice/volume.

## Results

**Fig. 4:**
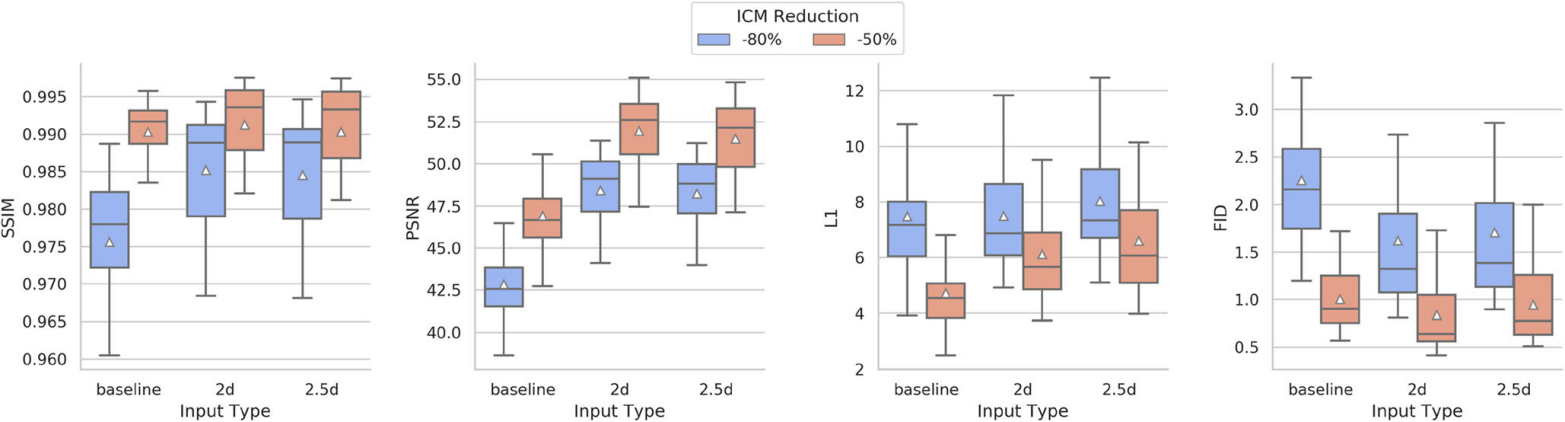
Scores and metrics for each model type (blue = −80%, red = −50%) as a boxplot. The calculation of the boxplots is based on the single volume scores from each ensemble model. The triangle within each boxplot symbolizes the mean of the respective input type for the relevant score or metric

Figure 4 contains the evaluation results of the four ensemble models, two different input types, and two different ICM reduction levels. In addition to the model results, we calculated a so-called baseline for both models. The baseline calculation uses the image slices with ICM dose computationally reduced by 50% or 80% and compares them to the full ICM dose image. With this, it is possible not only to compare the achieved results of the models to the target but also to track the improvement of the image similarity and quality in comparison to the input slice.

For each quantitative analysis score, an image improvement compared to the baseline image could be shown. In general, the networks that applied a 50% ICM reduction achieved substantially better image quality than the networks that applied an 80% ICM reduction.

The −80% models reached a mean SSIM of > 98%, PSNR of > 48, L1 of between 7.5 and 8, and an FID between 1.6 and 1.7. In comparison, the −50% models reached a mean SSIM of > 99%, PSNR of > 51, L1 between 6.0 and 6.1, and an FID between 0.8 and 0.95.

In addition to this quantitative image analysis, a qualitative image analysis using the VTT was also performed. The results of the VTT are listed in Table 2.

**Table 2:** Results of the VTT with three radiologists (A, B, C) for the different ICM reduction levels, models, and the slice/volume wise evaluation. Each model configuration is evaluated based on the real/fake accuracy and pathological consistency for single slices and complete volumes

		Volume						Slice					
ICM reduction	Models	Real/fake accuracy			Pathological consistency			Real/fake accuracy			Pathological consistency		
		A	B	C	A	B	C	A	B	C	A	B	C
−80%	2D	100%	59%	100%	23%	55%	32%	88%	90%	76%	85%	80%	90%
	2.5D	100%	100%	100%	27%	45%	45%	90%	88%	82%	92%	81%	90%
−50%	2D	95%	91%	95%	100%	100%	100%	69%	80%	62%	99%	98%	100%
	2.5D	73%	77%	91%	100%	100%	91%	55%	77%	64%	100%	100%	100%

In general, the results indicate, as expected, that it is more difficult for radiologists to distinguish between real and artificial images if they see only slices rather than a whole volume. However, the results of the VTT showed substantial differences between the 50 and 80% ICM reduction networks on the one hand and between the 2D and 2.5D networks on the other. Overall, the results of the networks with an 80% reduction were not satisfactory. On the one hand, these were easily identified by the radiologists as artificial due to artifacts, and, on the other hand, they did not show an excellent pathological consistency with the 100% ICM images (volume: 23%, 55%, 32%; slice: 85%, 80%, 90%). In contrast, the 50% reduction networks showed satisfactory results. These networks achieved 100% pathological consistency in the median both as 2D and as 2.5D networks in the volume analysis. Overall, however, the 2.5D networks were clearly more difficult to identify by the radiologists in the volume analysis than the 2D networks (95%, 91%, 95% vs. 73%, 77%, 91%).

**Fig. 5:**
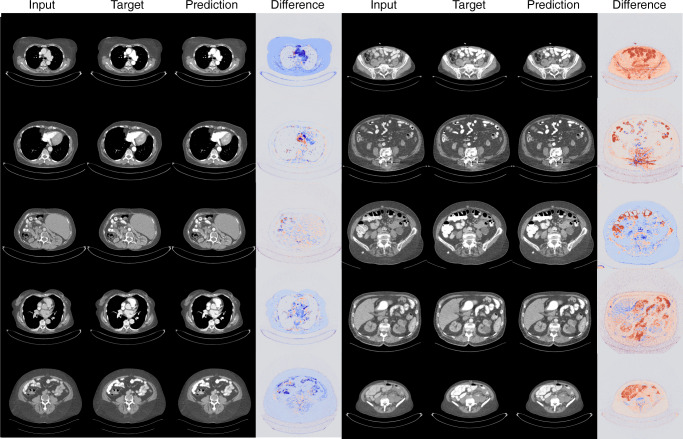
50% 2.5D ICM reduction network. Comparison of input, target, prediction, and difference image used and generated by the 2.5D model (−50%). The diffmap indicates the difference in HU between the target and the output images. The red regions indicate that the model predicted a lower ICM intensity (−50 HU) for a specific region, whereas blue regions indicate a higher ICM intensity (+50 HU) prediction for a region

**Fig. 6:**
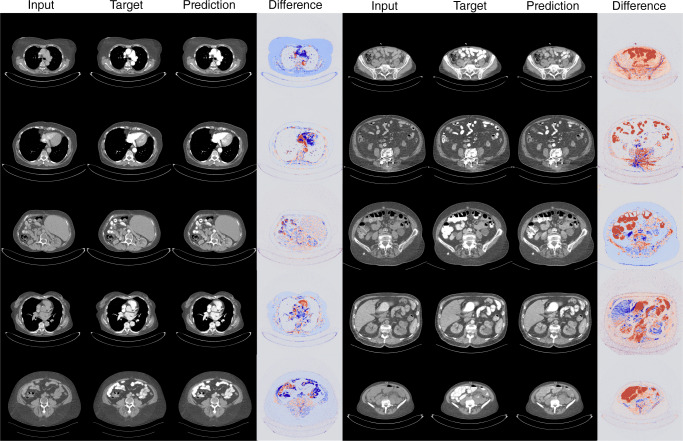
80% 2.5D ICM reduction Network. Comparison of input, target, prediction, and difference image used and generated by the 2.5D model (−80%). The diffmap indicates the difference in HU between the target and the output images. The red regions indicate that the model predicted a lower ICM intensity (−50 HU) for a specific region, whereas blue regions indicate a higher ICM intensity (+50 HU) prediction for a region

In addition to the presented results, Figs. 5 and 6 provide a visual overview of the models’ performance on selected CT slices.

## Discussion

In the present study, we trained generative adversarial networks to enhance ICM-induced image contrasts selectively and validated the results using dual-energy CTs in which a 50% or 80% reduced ICM dose was simulated via the proportional subtraction of the calculated iodine maps. To evaluate the network’s efficiency, we measured various quantitative parameters (L1 loss, SSIM, PSNR, FID) and performed a VTT with three radiologists.

Overall, our results show that the virtual enhancement of ICM is not only possible but also reached degrees in terms of quality; the generated CTs were pathological equivalent to the original ones. In general, the networks based on 2.5D performed slightly better in all quantitative evaluation parameters. However, in the volume-based VTT, they performed much better than 2D networks. This could be because scrolling through a volume created by the 2.5D network using the anatomical information creates a more natural flow than the 2D network. Furthermore, artifacts created by a 2D network may be more apparent in 3D data. However, the ICM dose was a much more relevant parameter.

All in all, the networks with 50% ICM dose reduction performed considerably better than those with 80% ICM dose reduction. This led to a median 100% consistency of pathologies in our test data of those images created by a network which inserts 50% ICM. This result is especially important regarding the possible insertion of pathologies described in the literature on unpaired image-to-image conversion [13]. Therefore, we conclude that a 50% ICM reduction is feasible and safe using GAN-based image-to-image translation. Overall, our results fit well into the recent literature, which has described the versatile possibilities of GANs for image processing, for example, in the insertion of contrast media in brain MRIs [14]. Further studies have shown that GANs are suitable as an alternative reconstruction algorithm to remove artifacts and reduce image noise in low-dose CT [15, 16].

As an alternative to our approach, the amount of contrast medium required on CT can be significantly reduced by using low-voltage technology. This technique enables to reduce the amount of ICM to a similar extent as with our approach [17, 18]. On the downside, the image noise is increased when using low-voltage CT [19]. This is especially important for bariatric patients for whom it is recommended to increase the tube voltage in order to reduce image noise [20]. This includes about 34% of all Americans [21] and is expected to increase to 42–51% of all Americans by 2030 [22].

In contrast, our method is not dependent on patient weight and can be implemented relatively easily and hardware-independently using post-processing. Furthermore, our approach may be used synergistically with low-voltage CT to even further reduce the dose of ICM. However, this needs to be verified by us in subsequent studies.

For the qualitative analysis of GANs within the field of medical imaging, the VTT is a widely used evaluation method for image quality through professionals [11, 12, 23]. For the setup within this study, a side-by-side approach was used to combine the real/fake selection with the question of pathological consistency. Within future studies, it would be worth also considering to conduct a fully blinded test scenario.

The most important limitation of our study is that, for ethical reasons, we were not able to conduct true double examinations with and without ICM dose reduction. Our workaround was to virtually reduce the ICM dose by calculating iodine maps from dual-energy CT examinations. While the low ICM dose studies calculated in this way looked very convincing to human radiologists, at this point, we cannot say with certainty that our GAN-based approach would also work in real ICM dose reduction regimes. Therefore, our next step is to reproduce our results first in a phantom and then in an animal model. Further limitations within this study are the used ICM reduction levels of 50% and 80%. Within further studies, it should be tested if there are other reduction levels that could reproduce or even reach better results within the reduction level range of 50 to 80%. In addition, it should be tested whether the perfect degree of reduction is dependent on the examined body region. Another limitation is the number of enrolled patients. Although this problem was addressed by internal cross-validation and multiple repetitions of the experiment, external validation including different scanner models and acquisition protocols and recruitment of larger multi-center patient cohorts should be the focus of future studies to demonstrate the true potential for contrast media insertion in CT and to validate it for clinical use.

In summary, it can be said that a GAN-based approach for subsequent selective contrast enhancement in CT examinations with virtual ICM dose reduction works excellently. However, future systematic phantom and animal studies still have to prove that this procedure also works with a real ICM dose reduction and which dose reductions can ultimately be achieved in clinical routine.

## Abbreviations

CI-AKI: Contrast-induced acute kidney injury
CIN: Contrast-induced renal injuries
CKI: Chronic kidney disease
FID: Fréchet inception distance
GAN: Generative adversarial network
HU: Hounsfield unit
HVS: Human visual system
ICM: Iodine-based contrast media
MAE: Mean absolute error
MSE: Mean squared error
PSNR: Peak signal-to-noise ratio
SSIM: Structural similarity index
VNC: Virtual non-contrast

## Declarations

### Guarantor

The scientific guarantor of this publication is Prof. Dr. Michael Forsting.

### Statistics and biometry

One of the authors has significant statistical expertise.

### Methodology

• retrospective

• experimental

• performed at one institution
